# Selective interstitial doxorubicin for recurrent glioblastoma

**DOI:** 10.1002/ccr3.2546

**Published:** 2019-11-19

**Authors:** Valerii Matcovschii, Dan Lisii, Valentin Gudumac, Stanislav Dorosenco

**Affiliations:** ^1^ Neurosurgery Department Institute of Neurology Institute of Neurology and Neurosurgery Chisinau Moldova; ^2^ Biochemistry Department Nicolae Testemiţanu State University of Medicine and Pharmacy Chisinau Moldova

**Keywords:** doxorubicin, glioblastoma, interstitial chemotherapy, recurrence, stem cell culture

## Abstract

Poor delimitation from the viable brain, great genetic heterogeneity, lack of tumor vascularisation are factors that preclude intravenous chemotherapy. Interstitial chemotherapy could be a strategy to avoid a blood‐brain barrier and to assure a minimal dose concentration of the chemotherapy agent. Doxorubicin might be devoid of neurotoxic effects and still efficient on remaining far‐infiltrated tumor cells.

## Graphical abstract

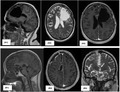

## 1 INTRODUCTION

The objective of this paper is to present two clinical cases of grade III and IV glioma patients that were treated with interstitial doxorubicin after undergoing two surgeries to remove a recurring tumor. Doxorubicin was tested in vitro in the primary cell culture that was obtained from the recurrent tumor. The patients presented with a recurrent frontal malignant glioma have undergone repeated surgeries. The drug was administered via a silicone catheter into the bed of the removed tumor in the interval between seven and 10 days postop, and both patients showed significant clinical improvements after three sessions of interstitial chemotherapy. After a month and a half, the first patient experienced sudden clinical decline which leads to a coma and ultimately, a lethal outcome. The morpho‐pathological analysis found small hemorrhagic lesions in the thalamus. There are no signs of the tumor recurrence in the second patient, 2 months post‐treatment. Doxorubicin could be an effective drug for treating glial brain tumors both in vitro and in a clinical setting. An interstitial chemotherapy with doxorubicin is possible if all the methodological steps are properly undertaken. In addition to that, micelles or liposomes need to be tested as potentially neurotoxicity‐free forms of doxorubicin.

Glioblastoma is the most malignant tumor of the brain, characterized by high recurrence rates and a lethal outcome within 1.5‐2 years after surgical removal.

Several controlled studies which focused on the survival rate and the length of a relapse‐free period indicated a lack of statistically significant improvements in patients that were administered the latest chemo and radiation therapy after surgery. Most commonly, the chemotherapy combination used is temozolomide, carmustine, and avastin. Currently, selective interstitial chemotherapy after the tumor removal entailing the implantation of a slowly absorbable carmustine wafers is gradually gaining interest.^14^

Selective in situ chemotherapy, also called a convection method, is rarely proposed. In our cases, the drug was delivered via a silicone catheter into the bed of the removed tumor in the first seven to 10 days postsurgery. Substantial downsides of the systemic chemotherapy for case as of glioblastoma are as follows:

There is no evaluation of the sensitivity of the glioblastoma cells to the specific chemo treatment being administered.
Systemic chemotherapy does not meet the requirements of selective targeting of glioblastoma cells and instead it attacks the entire immune system and the rapidly dividing intestinal epithelium cells, myelo and lymphopoietic bone marrow cells, gonads, and hair follicles.
The drug concentration in the tumoral tissue varies greatly, and it is noncytotoxic between the administration intervals. This method does not allow an effective dosage control in the tumors.
The drugs used during systemic chemotherapy are required to cross the blood‐brain barrier.
This type of therapy is conducive to the appearance of drug‐resistant glioblastoma cloned cells, which need to be targeted with a much higher chemotherapy dose.

As opposed to a systemic approach, selective interstitial chemotherapy has a number of advantages:

The highest concentration of the chemotherapeutic agent is delivered directly into the bed of the remaining tumor.
When compared to a systemic approach, the dose of the administered drug decreases up to ten times, thus minimizing the potential of immunosuppressive side effects.
The choice of doxorubicin, which is a drug that does not pass the blood‐brain barrier, allows us to target the glioblastoma cells in a more precise manner, while avoiding systemic immunosuppressive and cytotoxic side effects.
An incremental administration allows for more frequent injections, which in turn allows to maintain the required concentration with less jumps.
Decreased risk of infection, as doxorubicin is an anthracycline antibiotic

## 2 CASE PRESENTATIONS

We present two female patients aged 46 and 64 (“A” and “B”, respectively), who were diagnosed with grade III and IV glial intracerebral tumors of the frontal lobes.

Patient A (grade III glioma) undergone radiation therapy (60Gr) 1 month later. A CT performed after 4 months revealed a tumor recurrence with progressive growth. Clinically, she presented with partial Jackson seizures with secondary generalization, right‐sided leg hemiparesis and oligoaphasia.

**Figure 1:**
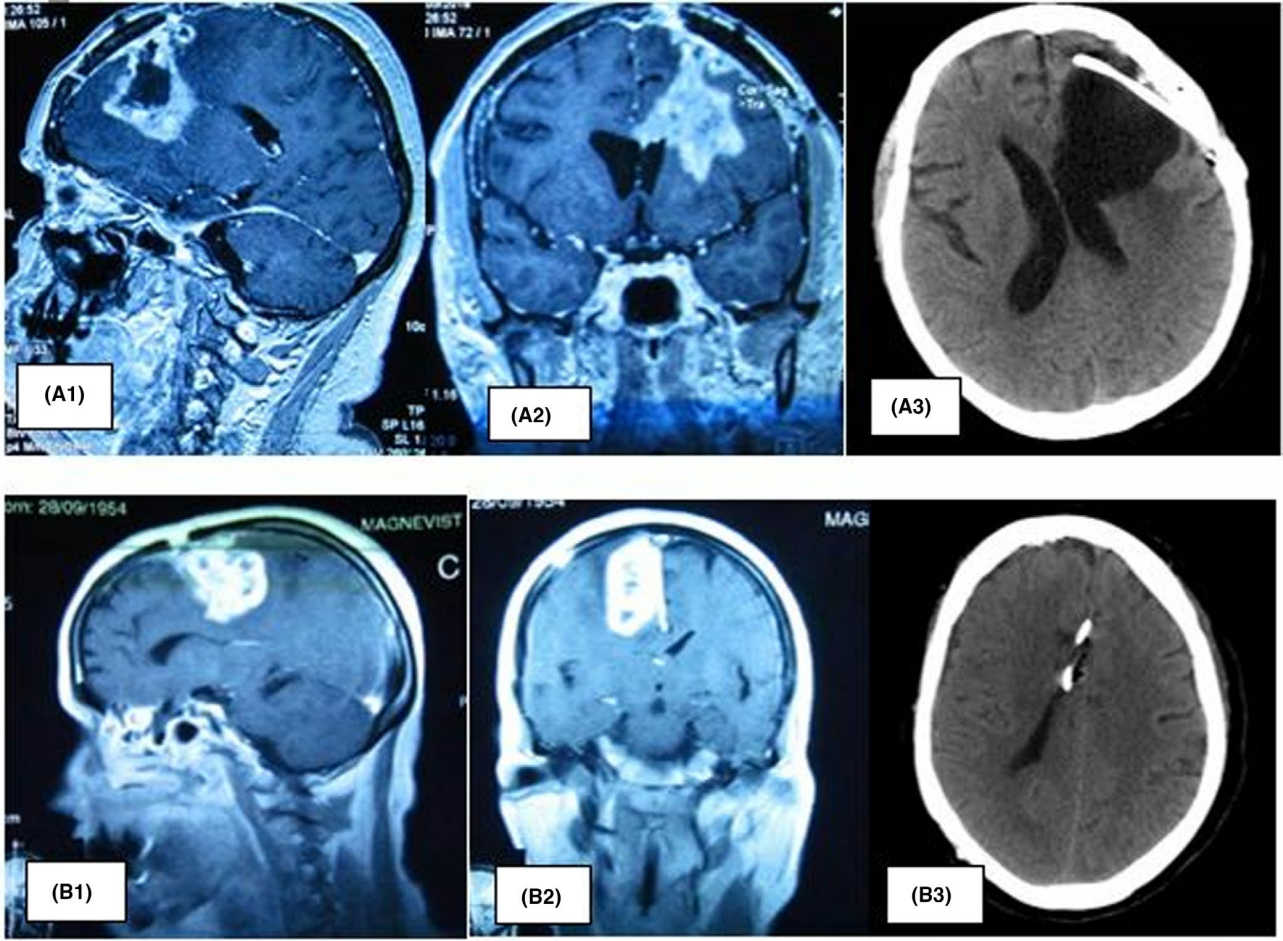
The removal of the recurrent glioblastoma with the insertion of the port. A1, A2, MRI patient A and (B1, B2) MRI patient B, glioblastoma recurrence 1 y after initial surgery. A3 and B3, CT scan of patient A and B, respectively, after gross total resection and insertion of the port‐a‐cath., in the bed of the tumor

The tumor regrowth was detected in patient B (grade IV glioma) after 3 months following the initial surgery, which then presented itself in the form of deep hemiplegia. Patient B was prescribed whole brain radiation therapy (Figure 1).

Both patients underwent another surgery, 1 year after the initial operation in the first case and only 5 months later in the second case. After obtaining approval from the ethics committee and getting the patients’ consent, a decision was made to perform selective interstitial chemotherapy following an internal protocol.

The operation of gross total resection was followed by subsequent installation of a “Celsite” port‐a‐catheter into the bed of the removed tumor (Figure 1).

## 3 METHODS AND MATERIALS

**Figure 2:**
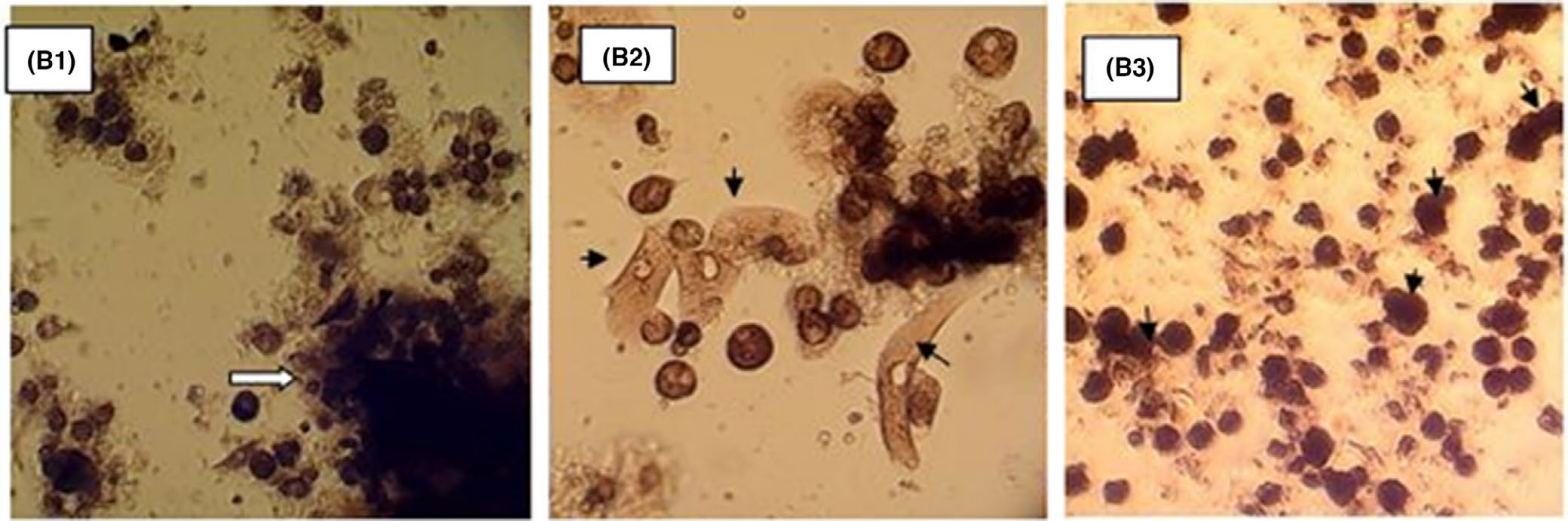
Initial glioblastoma culture isolated from patient B (G4) (magnification ×200). B1, cell culture 1 wk after cultivation (arrow indicate the initial fragment of the tumor). B2, fibroblasts seen in the cell culture after 2 wks (arrows). B3, the cell culture at 4 wks after three passage (the arrows indicate the mitoses)

A sample of the tumor that was removed during the surgery served for glial stem isolation, in order to perform chemo‐sensitivity testing to doxorubicin. Initially, the tumor is mechanically dissociated with surgical blades. Then, fragments are passed twice through 1.5 and 0.5 mm cell diameter filters, prior to them being seeded in DMEM glucose media with 20% serum (taken from the patient's blood), gentamicin, and fluconazole (100 μg/mL). The cells were grown at a 3%‐5% CO2 level, 85% humidity, and a temperature of 37 degrees Celsius (Figure 2).

**Figure 3:**
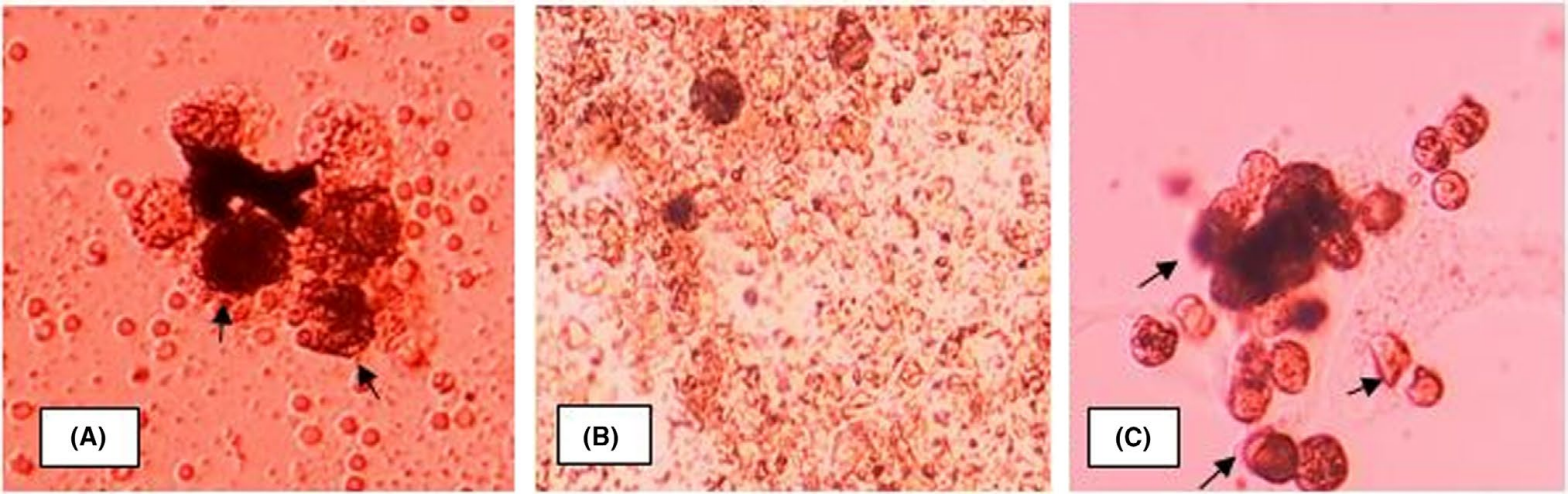
Glioblastoma sensitivity assay to Doxorubicin, patient B (magnification X200). A, 24 h after the injection of doxorubicin, cell damages shown by arrows (C = 0.002 mg/mL). B, An almost full glioblastoma cell lysis after 4 d since the addition of doxorubicin (C = 0.0002 mg/mL). C, Control cells, undamaged glioblastoma cells, day 7

After a period of growth lasting for 2‐3 weeks, the glioblastoma cells were selected through a several passages technique and then transferred onto a 24‐well cell culture plate. Each well contained anywhere from 50 up to 150 cells. Doxorubicin was added into each well at concentrations starting at 0.00002 mg/mL and up to 0.02 mg/mL. The cells status was verified microscopically at 1, 4, 24 hours, 3, 4, and 30 days. What was being taken into account were the number and the degree of damage of the glioblastoma cells depending on the exposure time to doxorubicin and the different concentrations. At 0.002 mg/mL, doxorubicin caused visible partial cell damage as early as a couple of hours; significant damage after 24 hours and full lysis after 3‐4 days. What is worth mentioning is that at lower concentrations (0.0002 mg/mL), the destruction of the glioblastoma cells starts later but reaches full lysis in the same 3‐ to 4‐day interval (Figure 3). In clinical settings, the recommended dose of doxorubicine administered intravenously is 25 mg/m^2^ per day, with the maximum amount per week reaching 100 mg. Considering the average volume of the removed tumor cavity, the sensitivity of doxorubicine in vitro and in order to avoid potential toxicity, the dosage that was used for the interstitial delivery was one of 0.1 mg/mL, which is ten times lower than the regular intravenous dose. The doxorubicin solution was prepared ex tempore, using 1 mg of the drug per 10 mL of water per injection.

Ten days after the second surgery, the patients were administered their first session of interstitial chemotherapy, with the first procedure carried out in the intensive care unit. The doxorubicin solution was injected through the port catheter using an electric syringe pump for thirty minutes, recorded on video and with the following metrics closely monitored: the patient's pulse, blood pressure, body temperature, neurological, and mental status. The patients were subjected to 3 sessions of 1 mg of doxorubicine introduced in the cavity of the removed tumor. The control CT performed after the 10 days of the procedure showed no signs of cerebral edema or any dislocation of the brain structures. Both patients were discharged in a satisfactory condition.

## 4 RESULTS

**Figure 4:**
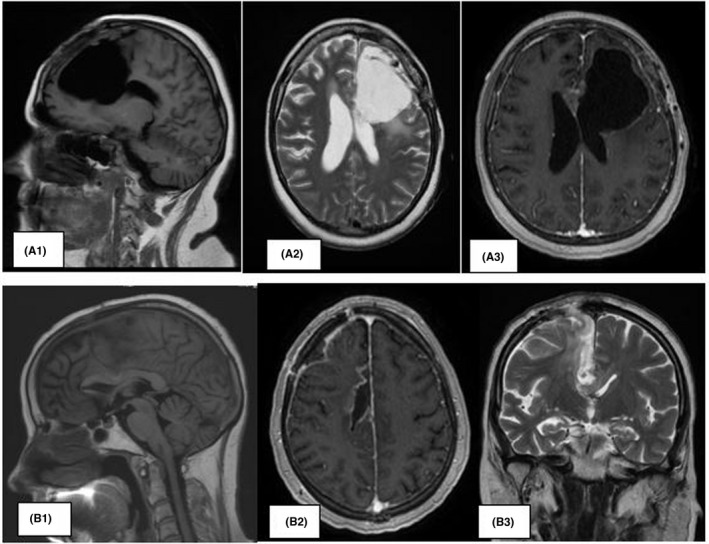
A1, A2, A3, MRI of patient A and (B1, B2, B3) of patient B, respectively, control after the second surgery and 3 sessions of selective interstitial chemotherapy via the port‐catheter

*Patient A*: A control MRI with a contrasting agent was performed on patient A 1 month after the second surgery, which showed a clear delimitation between the resected tumor and the brain tissue. No signs of the presence of the tumor were detected (Figure 4). Forty‐five days after the second surgery, patient A started experiencing severe headaches comparable to blows to the head and after a couple of hours, the patient became drowsy. The blood pressure dropped to 70/50 mm. HR. Art., the temperature dropped to 35.1 degrees C. The next day, the patient developed a coma (severity 1‐2), which lasted for 10 days, with a lethal outcome.

A pathological examination of the brain showed no macroscopic signs of the tumor. The findings comprised of massive glial necrotic changes to the brain tissue along the periphery of the removed cavity, as well as a small parenchymal hemorrhage in the posterior sections of the lateral walls of the third ventricle, over the mammary bodies in the posterior hypothalamus, around 10 mm in diameter. A histological examination of the areas of the cerebral cortex and partially white matter next to the border of the removed tumor revealed areas of massive necrosis and gliosis. An intense area of hemorrhage was found in the wall of the third ventricle in the hypothalamus region, with a large quantity of fibrins and lymphocytes.

*Patient B*: Clinical examination of the patient during the follow‐up was characterized by a general improvement and a gradually reducing hemiparesis. A dose of 1 mg of doxorubicin was administered to her once a week in an outpatient setting. A control MRI once month after the surgery did not show any signs of the tumor (Figure 4).

## 5 DISCUSSION

From a surgical point of view, the implantation of the port‐catheter in the bed of the removed tumor is a simple and safe procedure. One potential complication is meningoencephalitis, due to the presence of a foreign body (the catheter). However, it could be reduced by administering a solution of dioxine through the catheter. Doxorubicin itself is a powerful wide‐spectrum antibiotic that prevents the development and spread of infection. In the early implementation of the treatment with carmustine, the reported occurrence of postoperative infection was significantly higher.^3^, ^13^ The overall incidence of serious intracranial infection was 2.2%, more common in the Gliadel than in placebo arm (3.6% and 0.89%, respectively).

A slow delivery of doxorubicin with an electric syringe pump through the port‐catheter over the course of 30‐60 minutes did not result in clinical or vegetative reactions in the subjects. The lethal outcome in the first case occurred 45 days after the surgery and was caused by a small parenchymal hemorrhage in the posterior hypothalamus; which lead to distinct vegetative disorders, dramatic drops in blood pressure, hypothermia with the subsequent development of coma. One could explain such a delay in complications by a repetitive accumulation of the doxorubicin in the third ventricle, probably caused by the drain's proximity with the lateral cavity ventricle. Several reports showed that an inadvertently intrathecal use of doxorubicin caused disastrous effects on the spinal cord.^1^ In two cases, doxorubicin caused a severe myelitis with only a partial recovery. Still, in both cases the dose of doxorubicin was much higher, (×10) compared with the dose during interstitial delivery. 8, 11

Three sessions of such therapy did not result in cerebral edema, thoroughly verified by a CT control scan; on the contrary, we noted a faster reduction of the brain edema after the doxorubicin perfusion. Repeated blood tests did not reveal any systemic hematopoietic changes. The dose delivered was determined through the cytotoxic sensitivity of the glioblastoma cells following the tumor culture in vitro assay. The quantity of the doxorubicin introduced into the bed of the tumor per session was around 1 mg.

Doxorubicin is mostly indicated in lymphoma, leukemia, breast, and ovarian cancers, but has little clinical interest in cerebral malignant diseases.^11^ Several assays with doxorubicin in vitro, showed a quasi‐necrotic effect on glioblastoma cells with high selectivity at low doses.^10^ To date, there are no clinical data on doxorubicin administration in patients diagnosed with glioblastoma, either via systemic administration or interstitially. There are several essays on animals with promising results: significantly prolonging survival in bearing brain tumor in rats by delivering doxorubicin locally.^10^

Targeting vascular drug delivery through brain‐blood barrier (BBB) modification represents a promising strategy for the treatment of glioblastoma.^5^, ^7^ In canines and rodents, BBB modification revealed a good penetration of the doxorubicin in the cerebrum. Alternatively, no drug was detected in the absence of BBB modification.^5^ For the same study, neuropathological examination gave the evidence of necrosis and hemorrhagic infarcts, pointing out significant neurotoxicity of doxorubicin at doses as small as of 0.1 mg/kg. A low passage of doxorubicin through BBB (blood‐brain barrier) explains its high general toxicity and low intratumoral dose levels. Local interstitial delivery could avert systemic complications.^9^, ^15^

Some preferred doxorubicin forms are liposomes or micelles. The drug carrier system could provide neuroprotection effect, as stressed in assays with doxorubicin‐loaded monosialoganglioside micelles.^4^ Gangliosides (GM1) play neuroprotective and neurorestorative roles, as well as induce neurogenesis and promote cell differentiation.^2^, ^6^, ^14^

The same doxorubicin‐loaded micelles administered intravenously favor central nervous system doxorubicin accumulation. However, a preferential accumulation in central nervous system might not be enough to prevent systemic complications.^12^ The absence of patent vessels and infiltrative growth pattern of glial tumors represent a strong argument for local delivery versus systemic one.

## 6 CONCLUSION

Local interstitial doxorubicin could be clinically relevant in treating recurrent glioblastomas. Further clinical and experimental studies are needed to assert the role of interstitial chemotherapy using a port‐catheter after glioblastoma removal. Interstitial doxorubicin might address the main issue of glioblastoma recurrences by increasing the length of a relapse‐free period and prolonging the life of the patients. Micelles or liposomes should be tested as potentially neurotoxicity‐free forms of doxorubicin.

## CONFLICT OF INTEREST

No potential conflict of interest was reported by the authors.

## AUTHOR CONTRIBUTIONS

VM: involved in neurosurgical procedures and acquisition, analysis, interpretation of data of the work and revision. DL: involved in neurosurgical procedures and acquisition, analysis and interpretation of data of the work. VG: involved in stem cell isolation procedures and acquisition, analysis and revision of data. SD: involved in neurosurgical procedures and acquisition, analysis and interpretation of data of the work.
